# Transactions of the Fifteenth Annual Meeting of the Ohio State Medical Society

**Published:** 1861-07

**Authors:** 


ART. XI.—*Transactions of the Fifteenth Annual Meeting of the Ohio State Medical Society, held at Ohio White Sulphur Springs, June 12th, 13th, and 14th,* 1860. Columbus, 1860. pp. 267.

PROCEEDINGS of medical societies are much more acceptable in print than formerly, because they are so much more satisfactorily prepared. The volume before us, for instance, is a collection of facts and reports and addresses and the usual accompaniments of a medical convention, neatly contained in a rather attractive volume. Much of the interest attaching to all such associations and bodies is, of course, local. The address of Dr. L. Firestone, of Wooster, the President of the Society, is devoted to that never-failing subject for discourses, medical education. He is in favor of the six months' term, and does not seem to recognize the objections that have been urged against the extension of college sessions.

One improvement we will suggest in the next issue of the Society's "Transactions." No book is complete without an index or table of contents. So much is that omission felt, that in England a proposition was introduced in Parliament to fine any publisher or author who failed to insert so indispensable an addition. The contents of the work may be specified as follows:—

1. Report of Committee on Obstetrics, by M. B. Wright, M.D., Cincinnati; which embraces a large number of interesting physiological and practical facts and cases in that science and art. The writer expresses a decided repugnance to the use of pessaries, and discusses the views on prolapsus uteri, and other affections, embraced in the works of Professors Meigs, Miller, and others.

2. Report of the Committee on Cannabis Indica, by R. R. McMeans, M.D., of Sandusky. This paper sketches a history of the physiological effects, modes of preparation, etc., of this remarkable exotic, especially from the experience of the writer. It is mainly a collection of facts carefully culled from all practicable sources, though its applicability to certain pathological conditions—those, for instance, of a hysterical nature, sleeplessness, and nervous spasmodic cough—is based upon the confidence which Dr. McMeans reposes in its virtues.

3. Report of the Committee on Medical Literature, by Edward B. Stevens, M.D., Cincinnati. This is a review or *resumé* of the recent literary contributions of our countrymen, especially as regards Ohio medical literature. Excluding periodicals devoted to collateral or associate departments of medical science, there appear to be, at the present time, about thirty-five medical journals published in the United States, viz.: three weekly, eighteen monthly, eleven bi-monthly, and three quarterly. Fourteen States afford the medical journalism of the whole Union, if these statistics are perfectly reliable, and we have no reason for believing that they are not.

4. Report on the Effects of Chloroform upon the Intellectual Processes, by T. L. Wright, M.D., Bellefontaine. This paper is intended as "an inquiry concerning the credibility of testimony relating to transactions of a mind partly unconscious." In the view of the author, such evidence should be subjected to all the rules and exceptions of circumstantial evidence, and should be fully corroborated by other circumstances.

5. Report of the Committee on Obituaries, by C. P. Landon, M.D., Westerville.

6. Report of the Committee on Diseases of the Eye, by A. Metz, M.D.,

Massillon; though well written, comprises merely the views on ophthalmology of a general practitioner, who has not devoted special attention to the affections of the eye.

7. Report on Diseases of Cervix Uteri, treated with the local contact of Nitrate of Silver, by W. H. Reeves, M.D., Springfield.

8. Report of Committee on Medical Societies, by G. F. Mitchell, M.D., Mansfield, Ohio.

9. Report on Insanity, by Richard Grundy, M.D. A carefully prepared paper on mental affections, their causes, etc, the statistics of institutions, etc. A useful source for reference to those who are interested in a large class of unfortunates.

